# Dual-Target Additively Manufactured Electrochemical Sensor for the Multiplexed Detection of Protein A29 and DNA of Human Monkeypox Virus

**DOI:** 10.1021/acsomega.4c04460

**Published:** 2024-07-17

**Authors:** Luiz Ricardo G. Silva, Jéssica S. Stefano, Cristiane Kalinke, Robert D. Crapnell, Laís C. Brazaca, Luiz H. Marcolino-Junior, Marcio F. Bergamini, Craig E. Banks, Bruno C. Janegitz

**Affiliations:** † Laboratory of Sensors, Nanomedicine and Nanostructured Materials, Federal University of São Carlos, Araras 13600-970, Brazil; ‡ Institute of Chemistry, University of Campinas (Unicamp), São Paulo 13083-859, Brazil; § Faculty of Science and Engineering, Manchester Metropolitan University, Chester Street, Manchester M1 5GD, United Kingdom; ∥ São Carlos Institute of Chemistry, University of São Paulo, São Carlos, SP 13083-970, Brazil; ⊥ Chemistry Department, Laboratory of Electrochemical Sensors (LabSensE), Federal University of Paraná, Curitiba, PR 81531-980, Brazil

## Abstract

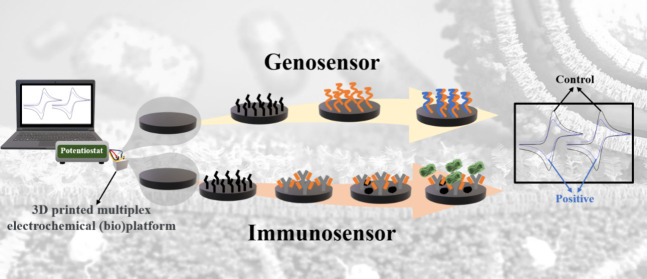

Herein, we present the first 3D-printed electrochemical portable biodevice for the detection of monkeypox virus (MKPV). The electrochemical device consists of two biosensors: an immunosensor and a genosensor specifically designed for the detection of the protein A29 and a target DNA of MKPV, respectively. The electrodes were manufactured using lab-made ultraflexible conductive filaments composed of carbon black, recycled PLA from coffee pods, and castor oil as a plasticizer. The sensors created through 3D printing technology exhibited good reproducibility and repeatability of analytical responses. Furthermore, both the immunosensor and genosensor demonstrated excellent MKPV detection capabilities, with a linear range from 0.01 to 1.0 μmol L^–1^ for the antigen and 0.1 to 20.0 μmol L^–1^ for the DNA target. The biosensors achieved limits of detection of 2.7 and 29 nmol L^–1^ for the immunosensor and genosensor, respectively. Interference tests conducted with the biosensors demonstrated their selectivity for MKPV. Moreover, analyses of fortified human serum samples showed recoveries close to 100%, confirming the absence of significant matrix effects for MKPV analysis. Therefore, the 3D-printed multiplex device represents a viable and highly promising alternative for on-site, portable, and rapid point-of-care MKPV monitoring.

## 1 Introduction

Still affected by the recent SARS-CoV-2 virus pandemic, humankind had to deal with the reemergence of the monkeypox virus (MKPV). Monkeypox is a zoonotic viral infection caused by MKPV.^1^ This disease causes skin lesions, fever, and headache among other symptoms, which appear within a few days after the infection and can lead to the hospitalization of the infected person.^2^ Although monkeypox has a low potential of becoming a pandemic, as the transmission of MKPV occurs mainly by direct contact with lesions or biological fluids of infected people or animals, the re-emergence of this virus raised concerns due to the increasing number of cases. Infections caused by MKPV have been reported in many nonendemic countries, including the United States and European countries.^3^ This attracted the attention of important health authorities around the world, including the World Health Organization (WHO).^4^ The recognition of viral diseases, including MKPV infections, is of paramount importance to estimate the extension of the disease, allowing fast decision-making by the health authorities, and controlling and monitoring the cases appropriately.^5^ In this regard, the development of simple, reliable, and fast methods for detecting MKPV plays an important role.

Electrochemical sensors have been presented as excellent and potentially applicable tools for the detection of viral diseases.^6,7^ The simplicity and high potential for application *in loco* make electrochemical sensors valuable platforms for the determination and quantification of viruses.^6,8,9^ Furthermore, electrochemical immunosensors and genosensors have gained great notoriety and popularity for being a simple and elegant form of diagnosis, with great potential for miniaturization and generating quick responses.^6,10−15^ The increasing use of additive manufacturing technology in the development of analytical platforms has provided great advantages in the development of electrochemical sensors, allowing the miniaturization and scalable production of analytical devices that can be excellent platforms for the biosensing of viruses, such as Hantavirus,^16^ Influenza^17^ and SARS-CoV-2.^18−20^

Additive manufacturing technology has enabled the application of high-performance lab-made conductive filaments for the production of complete electrochemical devices.^19,21−25^ Furthermore, the manufacturing of lab-made filaments can incorporate recycled polymeric materials, aligning with the principles of circular economy and sustainability.^22,23,26^ The fabrication of lab-made filaments using carbonaceous material and polymers like polylactic acid (PLA), whether recycled or not, has facilitated the production of various types of high-quality electrochemical biosensors for the detection of different viruses.^18,19,27^

In this context, Stefano et al., 2022,^19^ introduced a novel type of lab-made filament based on PLA and graphite for the 3D printing of sensors and biosensors. The produced biosensor was employed for detecting the S1 protein of the SARS-CoV-2 virus. Following the same target, Silva et al., 2023,^18^ developed a 3D-printed electrochemical immunosensor using lab-made filaments based on PLA and carbon black. Concerning lab-made filaments produced from recycled polymers, Kalinke et al., 2023^27^ created a lab-made filament from recycled coffee pods PLA, carbon black, and carboxylated multiwalled carbon nanotubes to develop a 3D-printed genosensor for yellow fever virus detection.

Recently, 3D printing has also made significant advancements regarding the conduction of analyses with multiple electrodes simultaneously, known as multiplex analyses. The development of 3D-printed multiplex electrochemical devices can be a powerful analytical tool for highly accurate clinical detections of multiple analytes, which include different biomarkers. In this sense, Morawski et al., 2023,^28^ created a versatile 3D-printed electrochemical device consisting of three working electrodes. The electrochemical device was employed to detect three different types of biomarkers: N protein, S_RBD_ protein, and anti-S_RBD_ from the SARS-CoV-2 virus. Therefore, 3D printing technology has successfully enabled the construction of a highly efficient multiplex electrochemical device for clinical analysis. In this way, multiplexed analyzes become a powerful analytical tool for virus detection tests, being able to confirm infection through more than one route, thus making the tests more reliable. Furthermore, they can also monitor possibly different infections simultaneously and different stages of the infection, opening up a new range of diagnoses.

Despite the great potential of 3D printing for creating multiplex systems, this technology has been poorly explored for this purpose to date. Additionally, besides the several types of electrochemical biosensors for virus detection that have been reported in the literature, to our knowledge, there is only one study reporting the detection of MKPV using an electrochemical sensor. Lima et al., 2023,^29^ developed a nanostructured paper-based biosensor modified with a CO_2_ laser for detecting the MKPV A29 protein. The electrochemical biosensor was able to successfully demonstrate the determination of the protein in human saliva and serum samples, thus demonstrating the potential applicability of an electrochemical sensor for MKPV detection. However, no type of 3D printed sensor or multiplex electrochemical systems for MKPV detection are reported in the literature. Thus, the development of 3D-printed multiplex platforms is relatively new, especially for MKPV detection, and is presented as an important field since these show great potential for improving clinical analyses, making it an extremely important research topic.

According to Stefano et al., 2023^5^ several specific analytes can be applied for the detection of MKPV, such as protein and genetic material. Among various proteins, I1L, M1R, and A29 are highlighted. The A29 protein, in particular, stands out for being one of the most common analytes for identifying MKVP,^30^ being highly conserved among poxviruses. It is a fusion protein present on the virus envelope that binds to cell surface heparin and is a target for neutralizing antibodies.^30,31^ Therefore, developing sensors specific for the A29 protein may allow direct detection of MKPV without the need for pretreatment of samples. Regarding the detection of genetic material, although it often requires extraction and amplification by PCR, its use is still extremely viable, being commonly applied by the main global disease control agencies (Test procedure: Monkeypox virus generic real-time PCR test). Moreover, genosensors provide highly specific and alternative methods to those already regularly used in analysis centers.^6,15^

Herein, we present new a 3D-printed multiplex electrochemical platform based on lab-made ultraflexible conductive filaments for the portable detection of recombinant protein A29 and a DNA target of the MKPV. The conductive filaments were produced from recycled coffee pods based on PLA, super P carbon black, and castor oil as a plasticizer. The multiplex platform consists of a two-working electrode system, in addition to counter and reference electrodes. To specifically detect MKPV, two different biosensors, an immunosensor, and a genosensor, were developed using the different working electrodes, and the analysis of MKPV was performed in human serum samples. The A29 protein was chosen because it is exposed on the surface of the virus and has a significant effect on its activity. Furthermore, this protein is highly conserved and the target of several neutralizing antibodies, which allows it to be detected directly in infected samples. Regarding the target genetic material (DNA), the sequence chosen was AAGCCGTAATCTATGTTGTCT. This target sequence is the product of a PCR protocol to detect MKPV recommended by the Centers for Disease Control & Prevention (Test procedure: Monkeypox virus generic real-time PCR test). Therefore, the development of a multiplex sensor to detect protein and genetic material can be a highly efficient alternative for reliable monitoring of MKPV.

## 2 Experimental Section

### 2.1 Reagents and Solutions

All chemicals used in this work were of analytical grade, and the solutions were prepared using ultrapure water with a resistivity higher than 18.0 MΩ cm from a Milli Q water purification system from Millipore (MA, USA). Potassium chloride (99 wt %), ferrocenemethanol (FcMeOH) (97 wt %), bovine serum albumin (BSA) from Fisher Chemical (Hampton, EUA), *N*-(3-(dimethylamino)propyl)-*N*′- ethylcarbodiimide hydrochloride (EDC) (98 wt %) and *N*-hydroxysuccinimide (NHS) (98 wt %), purchased from Sigma- Aldrich (St. Louis, USA), were employed in the construction and electrochemical evaluation of the immunosensor and genosensor. Human serum was obtained from Sigma- Aldrich (St. Louis, USA) and diluted in PBS 1x buffer at a factor of 100:1. Ethanolamine (>99 wt %, from Vetec) and PBS 1x were prepared from a mixture of sodium chloride (99% m/m, from Vetec) (137.0 mmol L^–1^), potassium chloride (2.7 mmol L^–1^), sodium phosphate dibasic (10.0 mmol L^–1^), and potassium dihydrogen phosphate (1.8 mmol L^–1^, from Vetec). For the immunosensor, a recombinant MKPV Protein A29 (antigen) and an MKPV A29-antibody (monoclonal) were used, which were acquired both from Sino Biological (Wayne, USA). For the genosensor, two DNA sequences were obtained from EXXTEND (Paulinia, Brazil): capture sequence (amino C6 – AGACAACATAGATTACGGCTT), target sequence (AAGCCGTAATCTATGTTGTCT) and the negative control sequence targets (TGACTACAGAAGTGGCTTTTG) and (TAGCCGGCAGCACAAGACATCT) from SARS-CoV-2 and Influenza A, respectively.

### 2.2 Apparatus and Electrochemical Measurements

All electrochemical tests were carried out using a portable μSTAT i-400 potentiostat (Metrohm DropSens, Spain) controlled by a computer with Windows 11 operating system (Intel core I5 processor and 8.0 GB RAM), using the Dropview 8400 software. All voltammetric analyzes were carried out in the presence of 1.0 mmol L^–1^ FcMeOH in 0.1 mol L^–1^ KCl. The responses for optimizations, construction of the analytical curve, and analysis of the samples were considered the difference in anodic peak current in the absence and presence of the antigen and target DNA. The responses were expressed as ΔI, considering the anodic peak current without any analyte (blank) minus the anodic peak current in the presence of analytes. A Sethi3D S3 3D printer (Campinas, Brazil) was used for printing the structures and electrodes, with the aid of the software Simplify 3D. A Filmaq3D extruder (Curitiba, Brazil) was used for the extrusion of the composites, producing the filaments. Scanning electron microscopy (SEM) analyses were performed using a Thermo Fisher Scientific model Prisma E equipment. Electrochemical characterizations were performed using the cyclic voltammetric technique. Fourier-transform infrared spectroscopic (FTIR) analysis was performed using a Tensor II (Bruker) spectrophotometer and the contact angle images were obtained using a lab-made apparatus by adding a drop of deionized water to the surface of the electrode.^32^

### 2.3 Recycled Filament Production and CB-rPLA Electrodes

The electrodes employed in this work were additively manufactured from a lab-made composite filament composed of 65 wt % recycled PLA, 25 wt % carbon black (Super P, > 99 wt %) from Fisher Scientific (Loughborough, UK), and 10 wt % castor oil from Merck (Gillingham, UK), which was called CB-rPLA, obtained following previous work.^33^ The electrochemical cell was designed in the form of a “box” with a square lid containing 4 independent entrances for each electrode: the working (2), reference, and counter electrodes. Figure S1 presents a schematic representation of the complete electrochemical cell. The base of the electrochemical cell was printed using the nonconductive polymer acrylonitrile butadiene styrene (ABS) from 3DFila (Brazil), and designed to have an internal volume of 1 cm^3^, requiring only 500 μL of solutions per analysis. The electrodes were designed in the shape of a “piston”, which makes it possible to fit into the electrochemical base cover easily and firmly. The electrodes were 1.5 cm high, with an analysis surface of 5.0 mm^2^ in diameter, and a total area of 19.6 mm^2^. The sensors were printed with a nozzle diameter of 0.6 mm, an extrusion temperature of 220 °C, a table temperature of 90 °C, a layer height of 0.1 mm, complete fill and printing speed of 1200 mm/min. To prevent the solution from coming into contact with parts of the electrode other than the surface, it was completely isolated with colorless, nonconductive nail polish. All sensors were polished with 1200-grit sandpaper to completely homogenize the surface and ensure their reproducibility.

### 2.4 Immunosensor and Genossensor Preparation

To prepare the immunosensor, antibodies anti-A29 MKPV protein were covalently bonded to the working electrode 1 (WE1) surface. For that, 20 μL of a solution containing 10.0 and 20.0 mmol L^–1^ EDC:NHS, respectively, in PBS 1× (pH = 7.4) were added to the electrode surface for 60 min. This process was followed by 60 min immobilization of 1.0 μg L^–1^ antibody MKPV (20 μL) in PBS 1 × (pH = 7.4). The specific antibody anchoring step on the sensor surface was fully optimized by univariate experiments. To this end, the deposition time and antibody concentration were optimized, ranging from 30 to 150 min and 0.5 to 20.0 μg L^–1^, respectively. For the last step in the construction of the immunosensor, 20 μL of a BSA solution (1% w/v) in PBS 1x (pH = 7.4) was added and incubated for 30 min to block any interaction sites available in the CB-rPLA. The electrode was rinsed after each step with PBS 1× and dried in air. After that, the immunosensor was ready for the detection of the MKPV.

**Figure 1:**
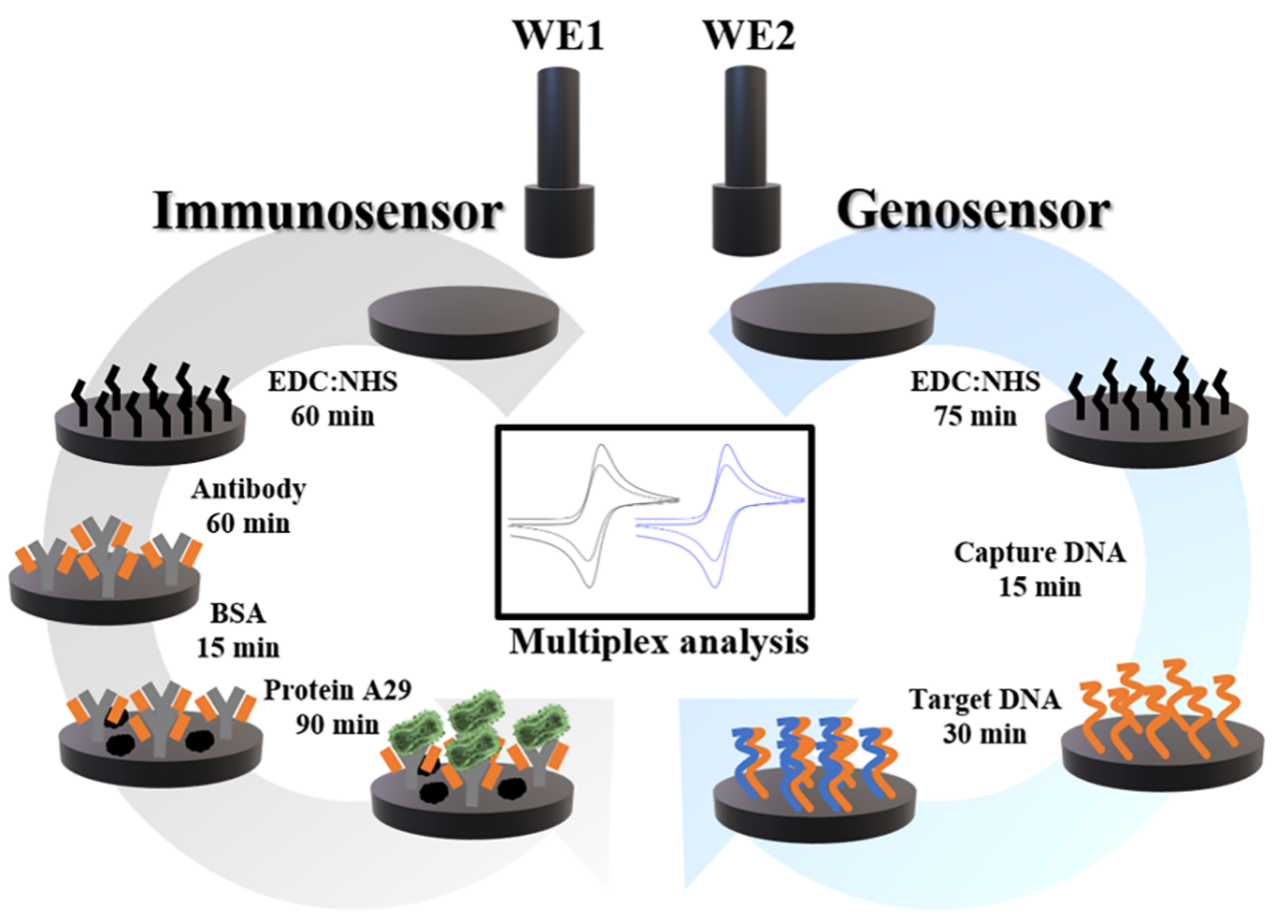
(a) Illustrative diagram of the biosensors preparation steps. (WE1 - immunosensor) step 1 - EDC:NHS immobilization; step 2 - anchoring of the specific antibody; step 3 - blocking with BSA and step 4 - label-free detection of the specific MKPV antigen. (WE2 - genosensor) step 1 - immobilization of EDC:NHS; step 2 - anchoring the capture DNA together with blocker/spacer; step 3 - hybridization with MKPV target DNA for label-free analysis.

To prepare the genosensor on the second working electrode (WE2) surface, a mixture of EDC:NHS in the same previous concentration was used, but with a time of 75 min. Then, a solution containing the capture DNA at a concentration of 6.0 μmol L^–1^ and the blocker/spacer ethanolamine at a concentration of 0.12 mmol L^–1^ in PBS 1× (pH = 7.4) was incubated for 15 min. The capture DNA immobilization step was also optimized, varying the time and concentration from 15 to 105 min and 1.0 to 10.0 μmol L^–1^, respectively. An illustrative scheme of each step can be seen in Figure 1. Furthermore, a time-lapse of the sensors manufacturing and preparation of the biosensors can be obtained in the Supporting Information.

## 3 Results and Discussion

**Figure 2:**
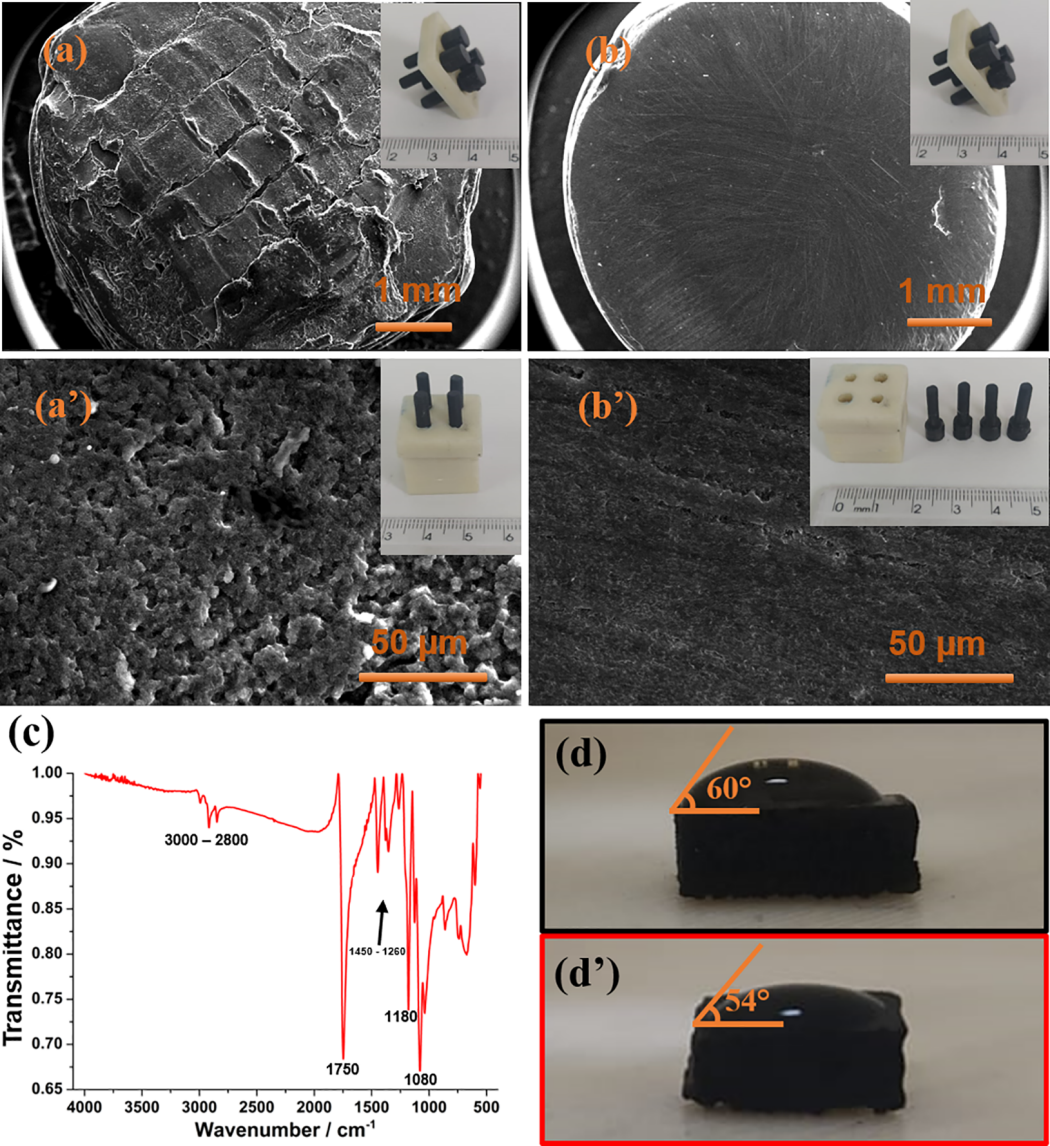
SEM images of the electrode surface (a) before polishing and (b) after polishing at different magnifications. (a-b) 31× and (b-b’) 1000 ×, respectively. (c) FTIR spectra for CB-rPLA, and (d and d’) water contact angle measurement for unpolished and polished electrodes, respectively. Inset: real images of the 3D printed multiplex system.

The production of 3D-printed sensors using flexible conductive filaments may exhibit some printing patterns at the fabricated electrodes. To form a smoother and reproducible surface, all produced electrodes undergo a thorough polishing process to homogenize the surface, ensuring enhanced reproducibility of analytical responses. A comparison of the surface morphology before and after the polishing process can be seen through the SEM images presented in Figure 2. The as-printed electrode showed an irregular surface, as seen in Figure 2a upon magnification of 1000x (Figure 2a’), the sensor exhibits significant surface irregularity, indicative of potentially high roughness compared to the polished electrode. Since no specific treatment is applied, much of this roughness can be attributed to the rPLA coating on the filament, which is the predominant material within it. After the sensor’s polishing (Figure 2b) more uniform surface is observed across the entire area, and this behavior is likewise observed in Figure 2b’, after magnification. While the polished sensor may appear less rough, it is crucial to note that polishing not only provides a smoother surface but also is capable of removing the excess rPLA from the surface, enabling the generation of reproducible and improved analytical responses, as well reported in the literature.^34^ SEM images (Figure S2) were also acquired for the filaments used in the 3D printing process. As can be seen, the filaments produced are uniform, with an absence of air cavities inside them (Figure S2b). These images demonstrate a homogeneous filament production, with no areas lacking material fill, and adequate mixing of all the components.

In the FTIR spectrum (Figure 2C) several peaks between 1080 and 1800 cm^–1^ are observed, representing different bonds such as carboxylic, carbonylic, and oxygenated groups. These responses were expected in sensors produced from filaments composed of rPLA since such bonds come from the rPLA itself.^19,35,36^ Furthermore, it is important to highlight that carboxyl groups allow the direct and simple covalent binding of bioreceptors without the need for intermediates.^37,38^ In Figure 2d-d’ the water contact angle measurements images are presented. It is possible to observe that the surface has a hydrophilic characteristic (angle <90°), both on the unpolished and polished electrodes.^32^ The slight change in the contact angle values of the droplet on the polished electrode (from 60 to 54°) can be attributed to the possible removal of rPLA excess from the surface and exposure of the CB.^39^ This decay in the contact angle can be attributed to the greater presence of CB on the surface, since it is an amorphous material, contains oxygenated species throughout its structure, and has a large number of sp^2^ edge planes, which can provide hydrophilic characteristics.^39,40^ Therefore, it is possible to observe by a simple contact angle measurement whether the mechanical polishing was successful in removing excess rPLA and exposing a little more carbon black on the surface.

The voltammetric profile of the 3D-printed electrochemical sensors was characterized through cyclic voltammetry (CV) in the presence of a redox probe (1.0 mmol L^–1^ FcMeOH in 0.1 mol L^–1^ KCl). CV experiments were conducted on 10 distinct sensors to assess the reproducibility of their production using 3D printing technology. Figure S3 presents the voltammetric response of the 10 different sensors. In Figure S3a, cyclic voltammograms of the 10 distinct 3D-printed sensors are depicted, showing peak current values for anodic and cathodic processes measuring 43.5 ± 1.3 and 28.6 ± 0.8 μA, respectively. The separation between anodic and cathodic peaks (Δ*E*_P_) was 88 ± 1 mV, with oxidation and reduction peaks occurring at approximately +0.112 and −0.024 V, respectively. This behavior suggests a quasi-reversible process. Furthermore, these results indicate a high level of reproducibility in the production of electrochemical sensors, highlighting that the combination of 3D printing technology and lab-made filaments based on carbon black and recycled rPLA can yield high-quality sensors.

The electrochemically active area of the sensors was determined by performing CV measurements with a scan rate varying from 10 to 100 mV s^–1^ in the presence of 1.0 mmol L^–1^ FcMeOH in 0.1 mol L^–1^ KCl. Figure S4 presents the voltammograms obtained and the respective plot of peak current as a function of the square root of the scan rate. In Figure S4a, it is observable that as the scan rate increases, both the anodic and cathodic peak currents proportionally increase. Figure S4b shows the linear behavior between the peak current and the square root of the scan rate, with the curves exhibiting R^2^ values close to 1, indicating a process predominantly defined by the diffusion of the species. Considering these results, the electrochemically active area was calculated using the Randles-Ševčík equation for quasi-reversible processes.^41^ The electrochemically active area calculated was 36.7 mm^2^. This value is considerably higher than the geometrical area of the electrode (19.6 mm^2^), indicating a roughness of the surface, which can serve as active sites for the redox processes.

**Figure 3:**
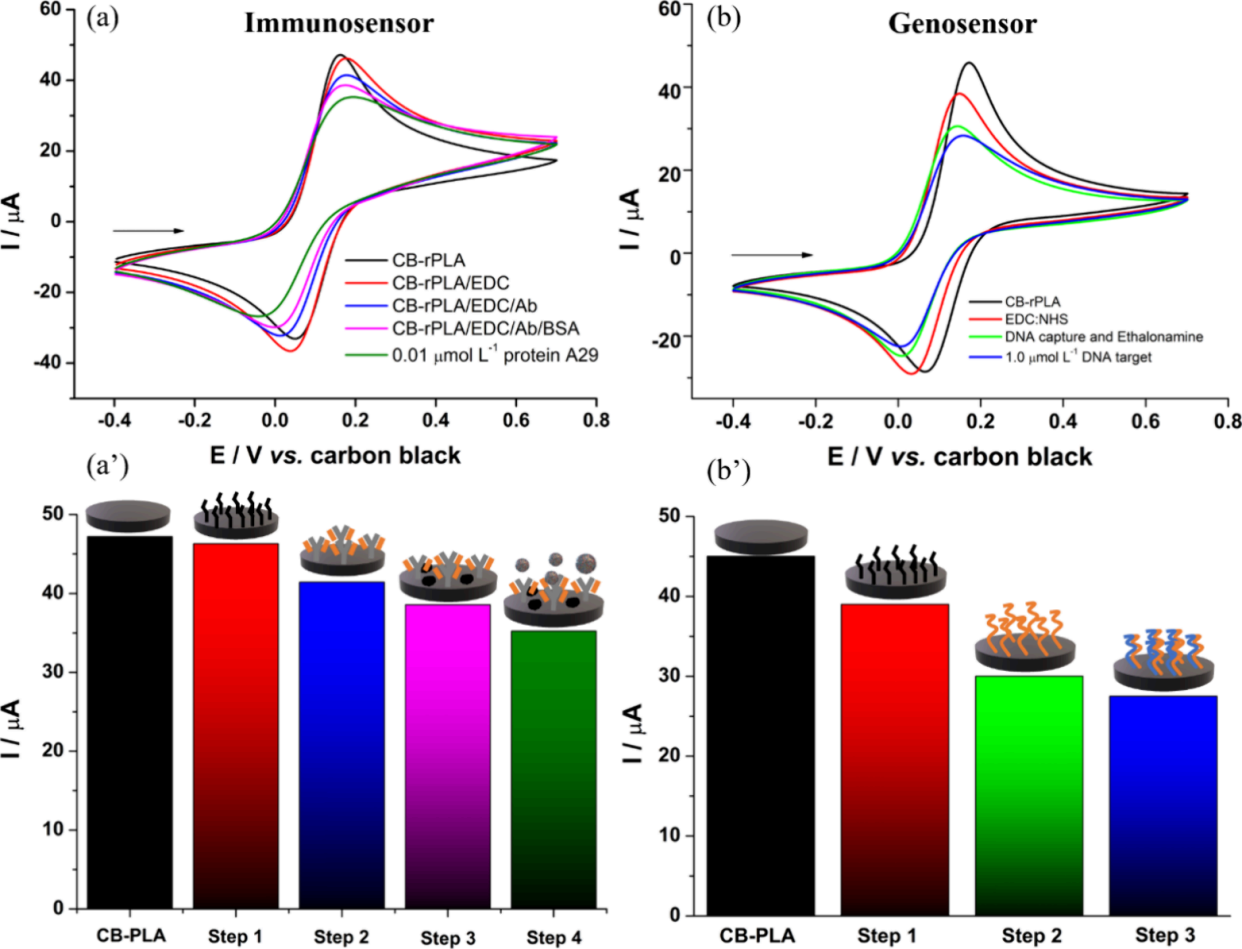
Cyclic voltammograms obtained with WE1 and WE2 in the presence of 1.0 mmol L^-1^ FcMeOH in 0.1 mol L^-1^ KCl. (a) Immunosensor steps: (black line) CB-rPLA; (red line) EDC:NHS; (blue line) Ab; (pink line) BSA and (green line) detection 0.01 μmol L^–1^ MKPV antigen. (b) Genosensor steps: (black line) CB-rPLA; (red line) EDC:NHS; (green line) DNA target + blocking and (blue line) detection 1.0 μmol L^–1^ MKPV target DNA. (a’) and (b’) bar graph of the anodic peak currents obtained in each stage of modification of the immunosensor and genosensor. Scan rate: 50 mVs^–1^.

The 3D-printed electrodes in this work are predominantly composed of rPLA, which is a carboxyl-rich polymer. These carboxyl groups are ideal for serving as ligands for EDC:NHS, subsequently allowing the production of highly specific electrochemical biosensors. Therefore, the sensor also eliminates the use of binding compounds to immobilize the EDC:NHS on the sensor surface, compounds such as cysteamine and glutaraldehyde, or metallic particles. In this context, it was possible to easily immobilize the anti-A29 protein antibody and the MKPV capture DNA on the proposed working electrodes of the 3D-printed electrochemical device since EDC:NHS is immobilized on the surface of the sensor by covalent bonding with carboxyl groups. Each step of the production of the immunosensors and genosensors for MKPV determination, was monitored by CV (Figure 3). For this purpose, 1.0 mmol L^-1^ of FcMeOH was employed as a redox. During each sensor modification step, a deposition of material occurred on the sensor’s surface. These layers partially “blocked” the sensor’s surface and reduced the analytical response in the presence of the redox probe. Consequently, it was possible to estimate the success of each step based on the decrease in anodic peak current observed in each stage.

In Figure 3a it is possible to observe that as the modification steps involved in the fabrication of the immunosensor (EDC:NHS; Ab, and BSA) are executed, the anodic peak current of WE1 exhibits a slight decrease. This decrease is evident in Figure 3a’, which presents the anodic peak current values obtained at each stage. This behavior is directly associated with the partial “blocking” effect caused by the compounds deposited on the surface. Furthermore, in the presence of the analyte (MKPV protein/antigen), a pronounced decrease in the anodic peak current also occurs, indicating that the developed immunosensor is capable of successfully detecting the MKPV antigen.

Regarding the genosensor developed on WE2 (Figure 3b), the same pattern can be observed, where the anodic peak current decreases as the modifications are performed (Figure 3b’), demonstrating the successful execution of the steps. Additionally, the sensor was able to detect the MKPV target DNA. However, the genosensor was prepared with one less step compared to the immunosensor, as the DNA capture anchoring step was carried out simultaneously with the blocking. To determine the effectiveness of performing these immobilization steps with both compounds simultaneously, we have also monitored the genosensor construction with the capture DNA anchoring and blocking steps separately conducted (Figure S5). It was observed that whether the steps were performed together or separately, there was no significant difference in biosensor performance with a current change (-ΔI) of approximately 5.0 μA for both tests. The etalonamine (blocking agent) is significantly smaller than BSA, another molecule used for this end, facilitating its insertion with the capture DNA on the electrode surface.^42^ Furthermore, this compound assists in the vertical orientation of the DNA strands on the sensor surface, facilitating their ability to hybridize with their complementary part.^43^ Also, there was not significant change in the signal due to the amount of material anchored on the surface of the sensor. Consequently, the decision was made to conduct this modification step together, as it reduces the time required for genosensor construction.

In addition to monitoring the modification and detection steps through the CV technique, the Electrochemical Impedance Spectroscopy (EIS) technique was also employed. Figure S6 presents the EIS results obtained at each stage of immunosensor and genosensor construction. It is observable that as the surface modification is carried out on WE1 (Figure S6a) and WE2 (Figure S6b), the charge transfer resistance (Rct) of the sensors increases. This increase is directly related to the partial blocking effect of the materials anchored on the sensor surface. In WE1, the Rct value for bare CB-rPLA was 40.1 Ω, while the value for the immunosensor fully constructed in the presence of 0.01 μmol L^–1^ antigen rises to 2231.2 Ω. In WE2, the initial value of Rct was 42.1 Ω, and after the hybridization with the target DNA, it increased to 2431.8 Ω. These results corroborate the findings from the CV study, demonstrating the successful construction of both biosensors and the detection of the target analytes.

After the construction of both biosensors, several optimizations were carried out to improve the performance of the platform. For that, cyclic voltammograms were recorded at each studied parameter using a 1.0 mmol L^-1^ FcMeOH solution. The concentration and modification time for the EDC:NHS anchoring and blocking steps were maintained fixed while the parameters related to the antibody on WE1 and the capture DNA on WE2 were optimized. Initially, the incubation time for Ab and capture DNA was studied. The deposition time for the Ab ranged from 30 to 150 min, and for the capture DNA, it varied from 15 to 105 min. Subsequently, the concentrations of these compounds were optimized. The concentration of Ab ranged from 0.5 to 20.0 μg L^–1^, and the DNA capture concentration varied from 1.0 to 10.0 μmol L^–1^. To monitor the efficiency of these modifications, all responses were obtained concerning the detection of the target analytes, at 0.5 and 10.0 μmol L^–1^ for MKPV antigen and target DNA, respectively. Figure S7 presents the responses obtained during the modification stages of biosensors WE1 and WE2 and Table 1 presents a summary of the studied parameters and chosen values.

**Table 1:** Summary of the Analytical Parameters of the WE1 and WE2 Biosensors

**parameter**	**immunosensor**	**genosensor**
linear range (μmol L^–1^)	0.01–1.0	0.1–20.0
slope (μA mol^–^^1^ L)	11.42	0.75
LOD (nmol L^–^^1^)	2.7	29
LOQ (nmol L^–^^1^)	9.3	89
RSD repeatability (*n* = 5)	4.18%	3.87%
RSD reproducibility (*n* = 5)	6.31%	5.63%

Regarding the concentration of Ab for the modification of WE1 (Figure S7a), the analytical response significantly increased at a concentration of 1.0 μg L^–1^ and stabilized at 5.0 μg L^–1^, with similar current levels for both. However, a significant decrease is observed at 10.0 and 20.0 μg L^–1^, which may be due to an excess of material deposited on the surface. The high amount of material during the modification may have hindered the response of the sensor. Consequently, a concentration of 1.0 μg L^–1^ Ab was chosen as optimal, as there is no significant difference between the response of 1.0 μg L^–1^ and 5.0 μg L^–1^ Ab. As shown in Figure S7a’, as the modification time with Ab (WE1) increased, the response in the presence of the virus antigen also increased. However, beyond 60 min, the improvement in response was not substantial, and the measurement error increased. Therefore, 60 min was considered the optimal modification time, as it provided a good analytical response and a relatively short modification time.

In Figure S7b it is possible to observe that as the concentration of capture DNA increases the analytical response increased until reaching concentrations up to 6.0 μmol L^–1^ and subsequently decreased. Therefore, the concentration of 6.0 μmol L^–1^ was chosen as optimal. In Figure S7b’, the increase in the modification time for capture DNA provided a decrease in the current response. This result is excellent as the shorter modification time yields the best analytical response. Thus, 15 min was chosen for the immobilization of the capture DNA. Therefore, for the construction of WE1, a 60 min modification time and 1.0 μg L^–1^ Ab were used for the immunosensor’s development, while for WE2, a 15 min modification time and 6.0 μmol L^–1^ of capture DNA were employed in the genosensor’s construction.

Following the optimization of the modification step with biorecognition compounds (Ab and capture DNA) on WE1 and WE2, the receptor-analyte binding time for detecting the target analytes was fine-tuned. For this purpose, the analyte recognition time was varied from 30 to 150 min for protein A29 and from 15 to 120 min for detecting the target DNA of the MKPV. Figure S8 presents the results obtained for both optimizations. For protein A29 recognition (WE1), the analytical response improved as the binding time increased up to 90 min. Thus, 90 min was chosen as the optimal time. In the WE2 sensor, the maximum analytical response was achieved at a hybridization time of 30 min and the response progressively decreased as the time was extended. Consequently, a 30 min hybridization time was chosen as optimal for the analysis of the target DNA of the MKPV virus.

**Figure 4:**
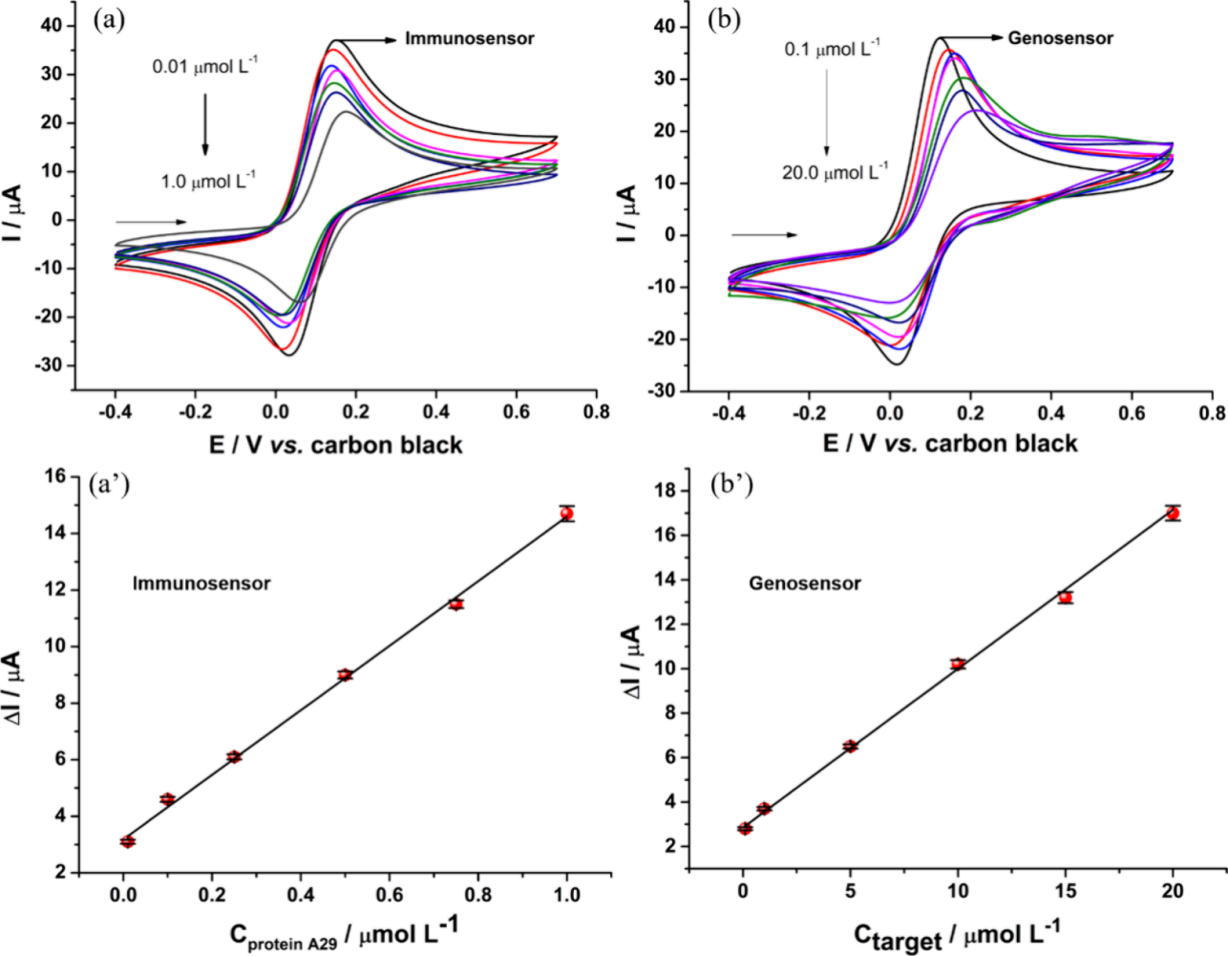
Cyclic voltammograms obtained with WE1 and WE2 sensors (a) immunosensor: varying the concentration of antigen from 0.01 to 1.0 μmol L^-1^; (b) genosensor: varying the concentration of target DNA from 0.1 to 20.0 μmol L^-1^. Calibration curves for the (a’) immunosensor and (b’) genosensor, obtained for variations in analyte concentration as a function of -ΔI.

After the optimizations were performed, two calibration curves were constructed to obtain the electrochemical immunosensor and genosensor analytical parameters. Concentrations of the antigen of 0.01; 0.1; 0.25; 0.5; 0.75 and 1.0 μmol L^-1^, and 0.1; 1.0; 5.0; 10.0; 15.0, and 20.0 μmol L^-1^ for target DNA were used to obtain the calibration curves for the immunosensor and genosensor, respectively. Furthermore, it is also important to highlight that small changes in the voltammetric profile may occur for the biosensor with its optimized constructions, when compared to the nonoptimized biosensors presented in Figure 3. Also, Figure 4 presents the results obtained for calibration curves constructed using both WE1 and WE2. As can be observed in Figure 4, as the analyte concentrations increase in both WE1 and WE2, the anodic current proportionally decreases. Consequently, it was possible to construct analytical curves based on the correlation between the concentration of protein A29 and MKPV target DNA. Both curves exhibited excellent linearity, with R^2^ > 0.99, demonstrating that within the proposed linear range, the biosensors can generate highly precise analytical responses. For WE1, the following linear equation was obtained: -ΔI (μA) = 3.18 + 11.42 × C_atigen_ (μmol L^-1^), while WE2 provided the equation: -ΔI (μA) = 2.85 + 0.75 × C_target_ (μmol L^-1^). The Limit of Detection (LOD) values were calculated based on the eq 3 × SD_intercept_/Slope, in which the LOD obtained for the immunosensor and genosensor were 0.0029 and 0.027 μmol L^-1^, respectively.

Reproducibility and repeatability tests were conducted for both immunosensor and genosensor against the analysis of 0.5 and 10.0 μmol L^-1^ of antigen and target, respectively. Reproducibility was evaluated through the construction of five distinct biosensors of each type. The results of the tests performed can be seen in Figure S9. The biosensors exhibited Relative Standard Deviation (RSD) values of 6.31% and 5.63% for the immunosensor (Figure S9a) and genosensor (Figure S9b), respectively. Regarding the repeatability test, the same biosensor was tested five times. For this, WE1 and WE2 sensors were modified to construct the biosensors, and the analyses of the target virus analytes were performed. Subsequently, the same sensors were polished until all deposited material on the sensors was completely removed, creating a renewed surface. With this, the biosensors were reconstructed, and the analysis was repeated to observe the repeatability of biosensor construction on the same sensor. This full process was repeated five times. The repeatability of the biosensors showed RSD values of 4.18% and 3.87% for the immunosensor (Figure S9c) and genosensor (Figure S9d), respectively. Therefore, the 3D-printed sensors presented a satisfactory surface for the production of the proposed biosensors with good repeatability and reproducibility, making them promising for biosensor construction. Table 1 summarizes the main analytical characteristics obtained for WE1 and WE2 sensors.

In the literature, some methods are used to detect MKPV, mainly spectroscopy and immunochromatographic assays, and also reports of electrochemical immunosensors.^5^ Lima et al., 2023^29^ developed an electrochemical biosensor from paper sensors manufactured with a CO_2_ laser and modified with gold nanostructures to detect the A29 protein. The biosensor had a LOD of 0.3 fg mL^–1^. Nevertheless, some methodologies based on other detection methods for the identification of this protein do exist. For example, Yu et al. (2022)^44^ reported an immunochromatographic assay coenhanced with Raman scattering colorimetry/surface for A29 protein detection. The authors reported the LOD of 0.2 and 0.002 ng mL^-1^ for the colorimetric method and surface-enhanced Raman scattering (SERS), respectively. In a parallel context, Wang et al. (2023)^45^ documented a dual-signal readout immunochromatography assay with colorimetric–fluorescence coenhanced capability for A29 protein detection. The methodologies presented LODs of 0.1 and 0.0024 ng mL^-1^. Moreover, Ye et al. (2023)^46^ employed an immunochromatographic test strip method for A29 protein detection, attaining a LOD of 0.05 ng mL^-1^.

Regarding the electrochemical genosensor, there are no reports of devices for detecting MKPV. Furthermore, a direct comparison of analytical characteristics with other works is impossible, as it is the first report in the literature of an electrochemical genosensor. However, in the literature, there is a wide range that describes DNA-based electrochemical sensors for detecting different types of viruses, such as SARS-CoV-2,^38^ SARS,^47^ yellow fever,^27^ Avian Influenza,^48^ Zika,^49^ among others. To detect these viruses, different types of electrodes were used, from more complex ones such as gold-coated plates to simpler and more practical ones such as screen-printed electrodes and 3D-printed electrodes. Regarding the LOD obtained, for Avian influenza a value of 10.0 pmol L^-1^ was reported, for SARS a value of 6.0 pmol L ^-1^, for Zika a value of 0.1 μmol L^-1^, for Yellow Fever of 0.138 μmol L^-1^, and SARS-CoV-2 a value of 0.3 μmol L^-1^.

In the present work, the 3D-printed immunosensor developed demonstrated a LOD of 30.7 ng mL^-1^ (2.7 nmol L^-1^). The genosensor obtained a LOD value of 29 nmol L^-1^, which can be considered “close” to that described in the present works (genosensors), demonstrating the effectiveness of the genosensor, even using a simple method, without the need for surface functionalization either, with gold particles or other components. Despite the LOD for the immunosensor being higher than reported in alternative techniques, it is imperative to underscore the inherent practicality in the development of electrochemical biosensors, the expeditious nature of analyses, and the relatively low cost. Additionally, the devised immunosensor and genosensor is entirely fabricated through 3D printing, ensuring prompt and decentralized large-scale production. Unlike other methodologies necessitating highly specialized operators and/or well-equipped laboratories, the method presented herein was entirely executed using a portable and user-friendly device, rendering it a point-of-care apparatus accessible to underserved populations.

**Figure 5:**
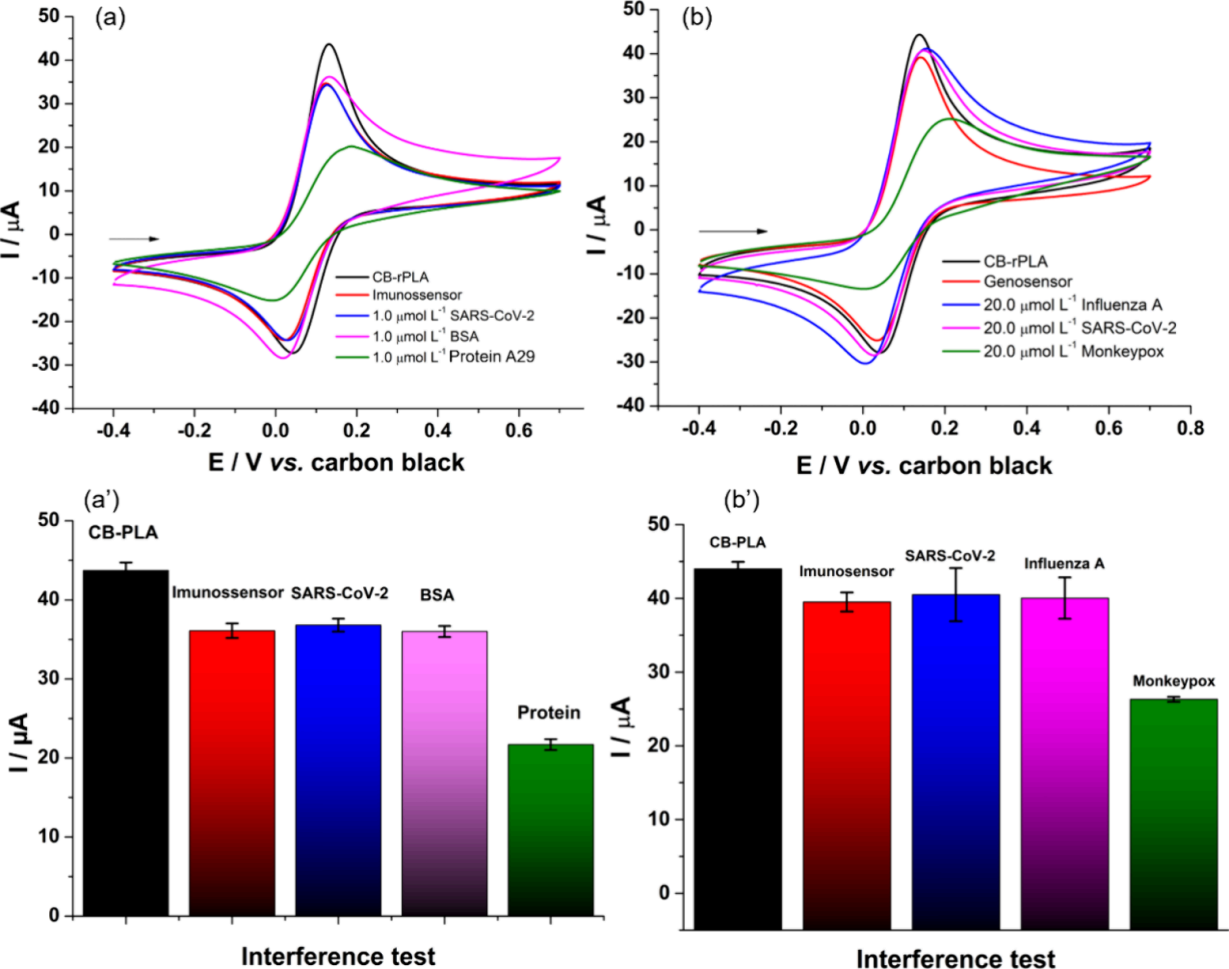
Cyclic voltammograms obtained with WE1 (a) and WE2 (b) for interference tests in the presence of 1.0 mmol L^-1^ FcMeOH in 0.1 mol L^-1^ KCl. (a’) (black line) CB-rPLA; (red line) immunosensor; immunosensor in the presence of (blue line) 1.0 μmol L^-1^ protein S1 SARS-CoV-2; (pink line) 1.0 μmol L^-1^ generic protein (BSA) and (green line) 1.0 μmol L^-1^ MKPV antigen. (b) (black line) CB-rPLA; (red line) genosensor; genosensor in the presence of (blue line) 20.0 μmol L^-1^ Influenza A target cDNA target; (pink line) 20.0 μmol L^-1^ SARS-CoV-2 target cDNA; and (green line) 20.0 μmol L^-1^ MKPV DNA target. (a’) and (b’) bar graph obtained from the anodic peak current of each analysis. Scan rate: 50 mV s^-1^.

To assess the specificity/selectivity of the constructed biosensors, they were tested against potential interferents (other viruses) commonly reported and highly infectious. For the immunosensor specificity test, the SARS-CoV-2 virus antigen and a generic protein (BSA) were used. For the genosensor, a cDNA fragment of the SARS-CoV-2 and Influenza A viruses were employed. The tests were conducted in the presence of 1.0 and 20.0 μmol L^-1^ of protein A29 and DNA, respectively, for each biosensor. Figure 5 presents the results obtained for all the conducted interference tests. As observed in Figure 5a-b, no significant voltammetric change is noted when the analysis was performed in the presence of interferents, both in WE1 and WE2. Furthermore, this behavior can be seen more clearly in Figure 5a’-b’, indicating that the anodic peak current remains unchanged in the presence of interferents. This behavior can be attributed to the specific biorecognition materials immobilized on the sensor’s surface, which have no affinity for nonspecific targets, only to MKPV antigen (WE1) and target DNA (WE2). Therefore, it can be inferred that the developed biosensors exhibit good selectivity and a high potential for specific MKPV detection.

Finally, to confirm the applicability of the manufactured biosensors, they were tested against the analysis of three human serum samples fortified with known concentrations of MKPV antigen (A29 protein) and target DNA. The fortified concentrations were 0.1, 0.5, and 1.0 μmol L^-1^ for the antigen and 0.1, 10.0, and 15.0 μmol L^-1^ for the target DNA. Based on the obtained peak current values, they were interpolated on the previously generated analytical curve, and the concentrations were determined. Figure S10 presents the results obtained in the analysis of the fortified samples and Table S2 presents a summary of the fortifications carried out and results obtained. In Figure S10a,b, it can be observed that as the analysis of the fortified samples was carried out, the anodic peak current decreased in proportion to the concentration present in the human serum sample. All the recovered concentrations (Figure S10a’,b’) were close to 100% of the originally fortified concentration, ranging from 92.1% to 104%. This result demonstrates that direct analysis can be performed on diluted human serum samples (100:1). Furthermore, recoveries close to 100% indicate that there is no matrix interference in the conducted analyses. Therefore, it can be inferred that the 3D-printed multiplex electrochemical bioplatform for the determination of MKPV antigen and target DNA is capable of monitoring the virus of interest practically and straightforwardly in human serum samples.

## 4 Conclusions

The 3D printing technology by FDM successfully allowed the production of a multiplex electrochemical device based on two working electrodes using ultraflexible lab-made conductive filaments, which were manufactured from recycled polymeric material. The modification of the working electrodes to obtain an immunosensor and a genosensor proceeded satisfactorily, allowing for the development of specific biosensors for different MKPV biomarkers. The biosensors exhibited a linear range of 0.01 to 1.00 μmol L^-1^ and 0.1 to 20.00 μmol L^-1^ for the immunosensor and genosensor, respectively. The achieved LOD values were 2.7 nmol L^–1^ and 29 nmol L^–1^ for the immunosensor and genosensor, respectively. Furthermore, selectivity tests were conducted for both biosensors against other viruses and a generic protein, demonstrating the biosensors’ excellent specificity for MKPV. Analysis of fortified human serum samples showed recoveries close to 100%, confirming the applicability of the multiplex device. Consequently, for the first time in the literature, a 3D-printed multiplex electrochemical device based on immunosensor and genosensor for MKPV determination is presented. Finally, the 3D-printed electrochemical device is highly qualified for the simple, practical, and portable determination of MKPV, making it suitable for on-site and point-of-care applications.

## Data Availability

The data are accessible through the Supporting Information associated with this research.

## Supporting Information Available

Video of the production of the 3D printed electrochemical device (MP4)
Design of the electrochemical device and its measurements; morphological images of the conductive filament; reproducibility of rCB-PLA electrochemical responses; electroactive area of rCB-PLA; voltammetric test for deposition of each individual stage of the genosensor; EIS of all stages of the immunosensor and genosensor; optimization of the biosensor construction; optimization of the interaction time between the receptor and the analyte; biosensor reproducibility and repeatability tests; result of the analysis of samples fortified with MKPV; tables of parameter optimization for sensor construction and sample recovery (PDF)
