# Supplementary material for: Dual-Target Additively Manufactured Electrochemical Sensor for the Multiplexed Detection of Protein A29 and DNA of Human Monkeypox Virus
Source: ACS Omega. 2024 Jul 17;9(30):33099–110. doi: 10.1021/acsomega.4c04460 (PMC11292847; doi:10.1021/acsomega.4c04460)
Supplement: Supplementary file 2 — ao4c04460_si_002.pdf [file ao4c04460_si_002.pdf]

Supporting Information: Dual-target additively manufactured electrochemical sensor for the multiplexed detection of protein A29 and DNA of human monkeypox virus

Luiz Ricardo G. Silva[1], Jéssica S. Stefano[1], Cristiane Kalinke[2], Robert D. Crapnell[3], Laís C. Brazaca[4], Luiz H. Marcolino-Junior[5], Marcio F. Bergamini[5], Craig E. Banks[3] and Bruno C. Janegitz[1*]

[1]*Laboratory of Sensors, Nanomedicine and Nanostructured Materials, Federal University of São Carlos, Araras, 13600-970, Brazil.*

[2]*Institute of Chemistry, University of Campinas (Unicamp), 13083-859, São Paulo, Brazil.*

[3]*Faculty of Science and Engineering, Manchester Metropolitan University, Chester Street, M1 5GD, United Kingdom.*

[4]*São Carlos Institute of Chemistry, University of São Paulo, São Carlos, SP 13083-970, Brazil.*

[5]*Chemistry Department, Laboratory of Electrochemical Sensors (LabSensE), Federal University of Paraná, Curitiba 81531-980, PR, Brazil*

*Corresponding author: brunocj@ufscar.br

Table of Content: **S1: Experimental**

Fig. S1. Design of the 3D printed multiplex device and its respective dimensions.

S2: Result and discussion

Fig. S2. Images obtained with SEM of different parts of the conductive filament and their respective amplifications

Fig. S3. Reproducibility test of the analytical response of ten different 3D printed sensors in the presence of an electrochemical probe.

Fig. S4. Test to calculate the electroactive area by varying the scanning speed from 10 to 100 mV s in the presence of an electrochemical probe.

Fig. S5. Analytical response of each genosensor modification step individually.

Fig. S6. EIS analysis of each modification step of the different biosensors constructed.

Fig. S7. Step of optimizing the concentration and modification time of the antibody for the immunosensor and capture DNA for the genosensor.

Fig. S8. Step of optimizing the deposition time of the antigen and target DNA analytes for interaction with their respective receptors in the biosensors produced.

Fig. S9. Reproducibility and repeatability tests of biosensors produced in the presence of the proposed anlytes.

Fig. S10. Analytical responses from analyzes of spiked blood and saliva samples.

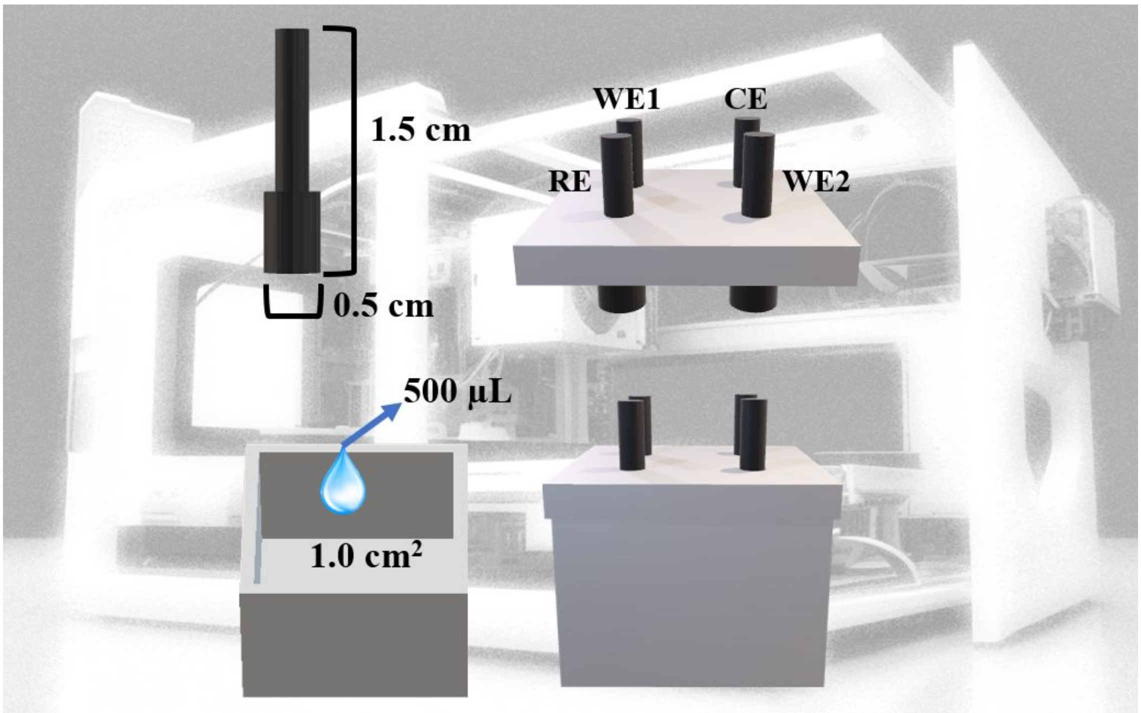

Fig. S1. Design of the multiplex system manufactured entirely in 3D

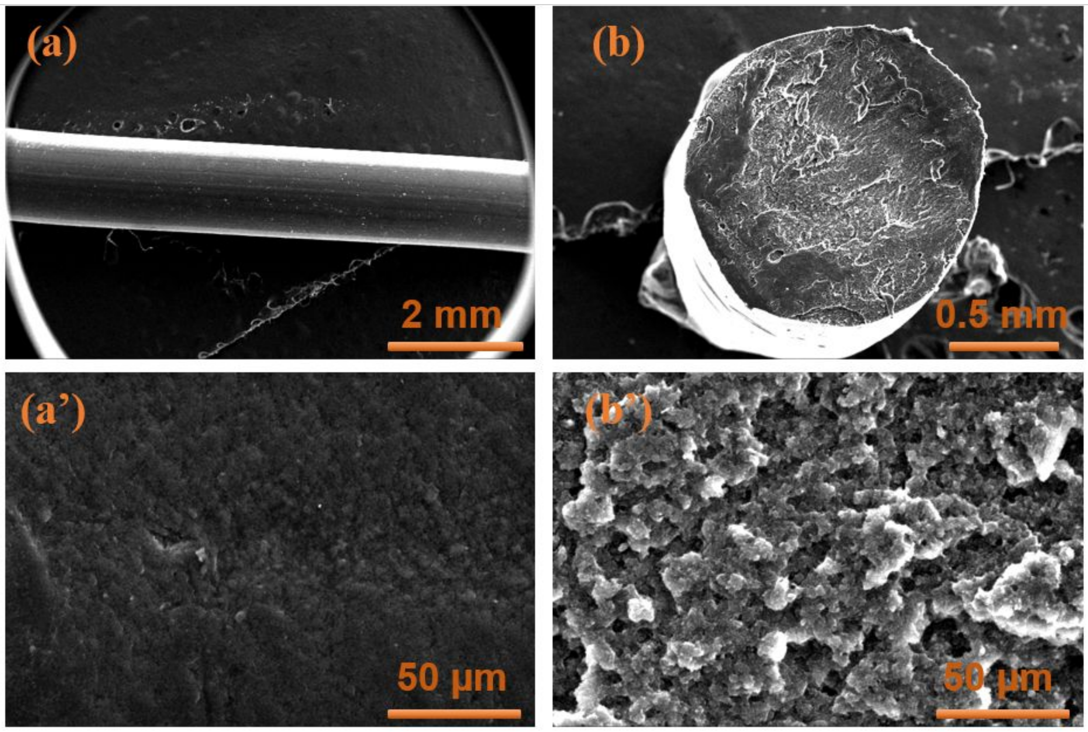

Fig. S2. SEM images of the filament surface (A) lateral view (B) sectional cut view at different amplification magnitudes. (A-A' and B'-B')

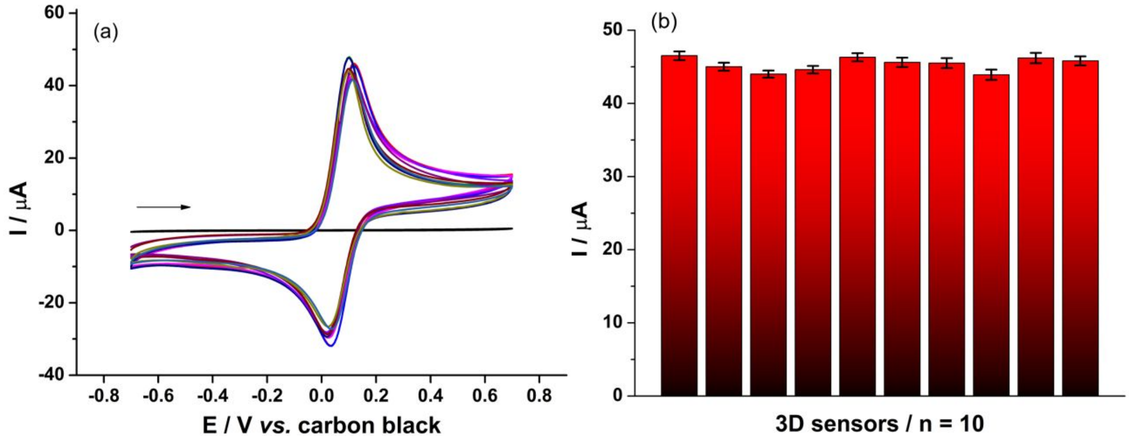

Fig. S3. (a) Cyclic voltammograms recorded using different electrodes (n = 10) in a solution containing 1.0 mmol L^{-1} FcMeOH in 0.1 mol L^{-1} KCl. Scan rate: 50 mV s^{-1}. **(b)** Bar graphs for anodic peak current values of different sensors in triplicate (n = 3).

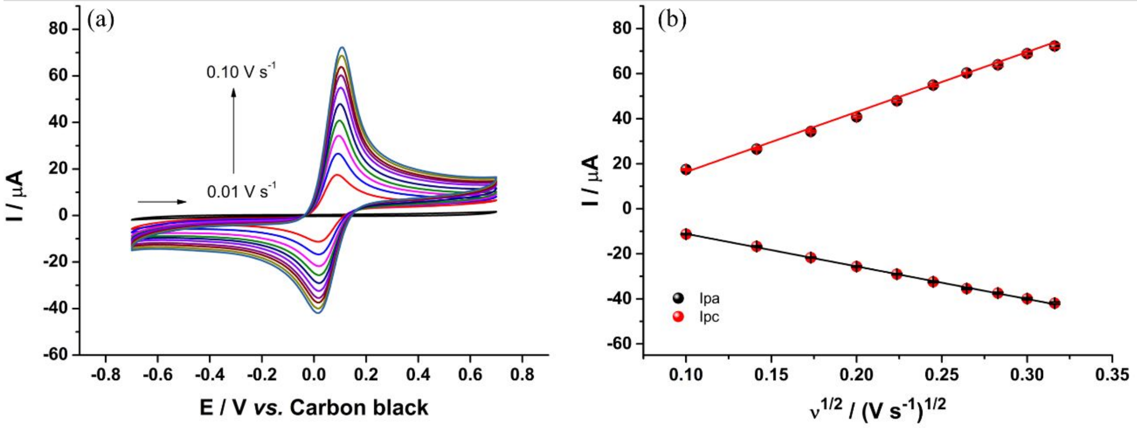

Fig. S4. (a) Cyclic voltammograms recorded in a solution containing 1.0 mmol L^{-1} FcMeOH in 0.1 mol L^{-1} KCl, at increasing scan rate values (10 to 100 mV s^{-1}). **(b)** Plot of peak current as a function of the square root of scan rate.

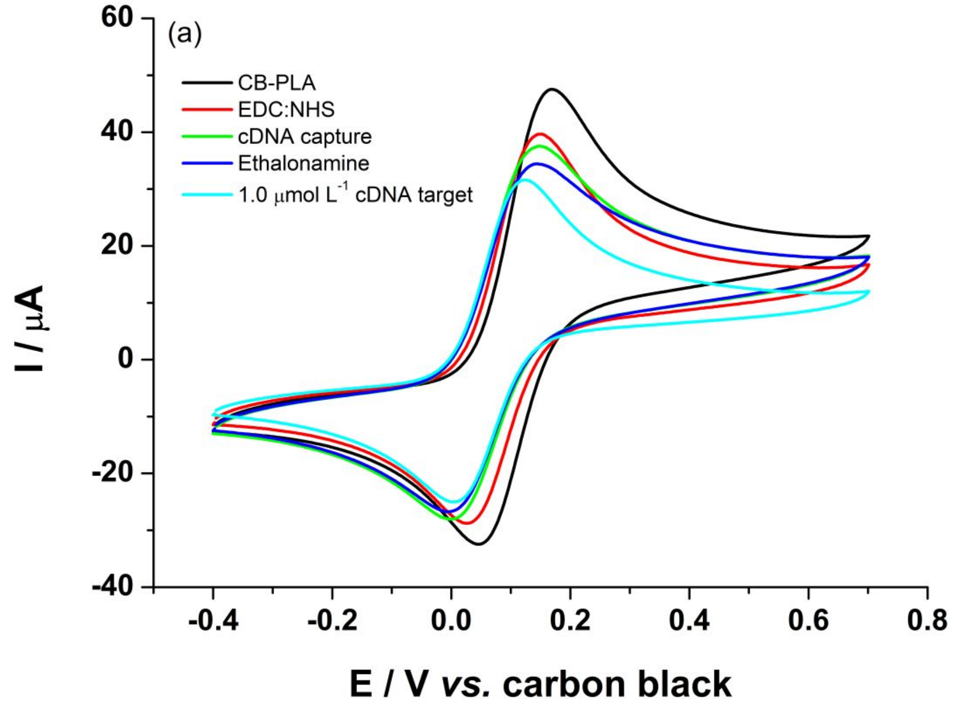

Fig. S5. Cyclic voltammograms obtained for construction **of** the genosensor (**black line**) CB-rPLA; (**red line**) EDC:NHS; (**green line**) capture DNA; (**blue line**) ethalonamine; (**cyan line**) 1.0 µmol L^{-1}. Analysis performed in the presence of 1.0 mmol L^{-1} FcMeOH in 0.1 mol L^{-1} KCl. Scan rate 50 mV S^{-1}.

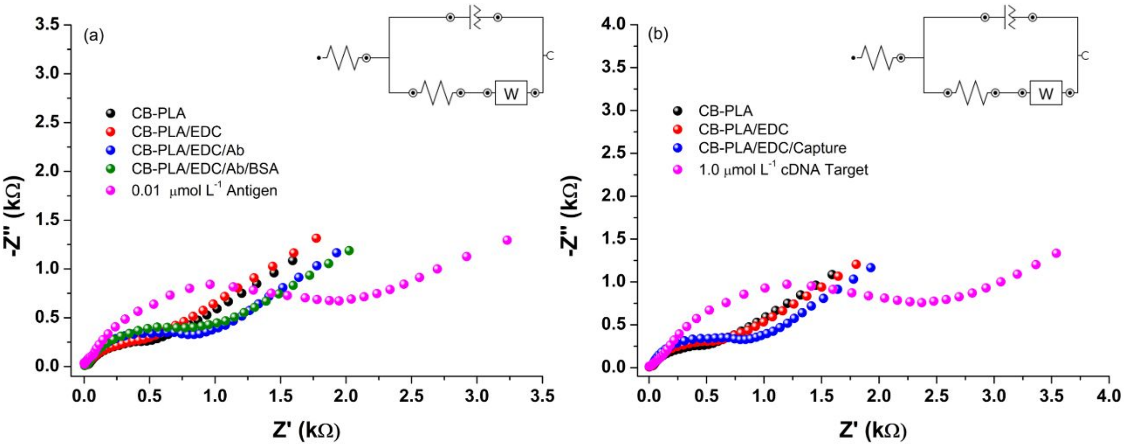

Fig. S6. Nyquist graphs obtained in the construction of (a) immunosensor and (b) genosensor. Inset: equivalent circuit employed. Applied potential of 0.08 V

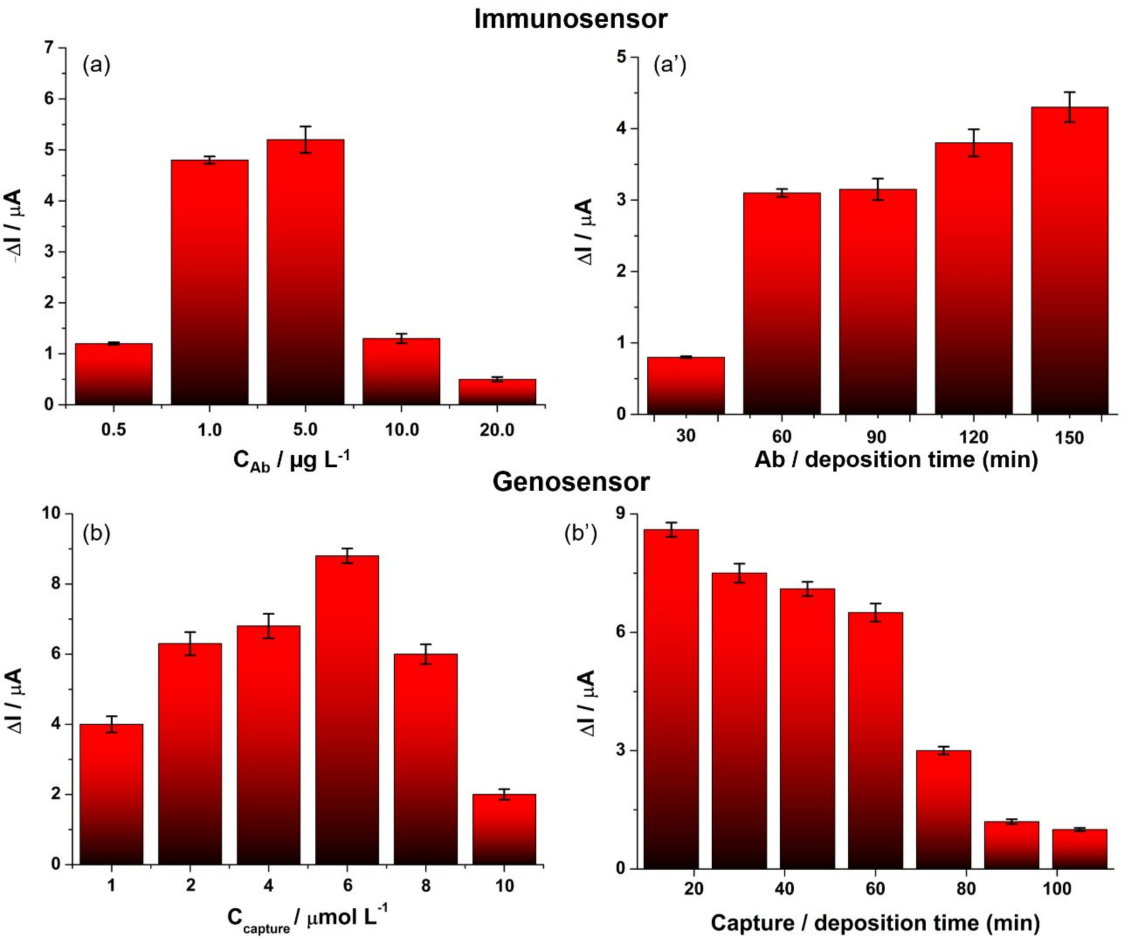

Fig. S7. Bar graphs of the responses to the optimizations of the biosensor modification step with antibody and capture DNA of the MKPV. (a-a) optimization of the

concentration (0.5 to 20 µmol L^{-1}) and interaction time (30 to 120 min) of the antibody on the surface of the immunosensor (WE1). (b'-b') optimization of the concentration (1.0 to 10.0 µmol L^{-1}) and time (15 to 105 min) of the DNA capture on the genosensor surface (WE2). Responses obtained as a function of the electrochemical response in the presence of 1.0 mmol L^{-1} FcMeOH in 0.1 mol L^{-1} KCl. Electrochemical response obtained after analyzing of the 0.5 and 10.0 µmol L^{-1} antigen and target DNA. Scan rate 50 mV s^{-1}. Responses obtained from the variation of the anode peak current.

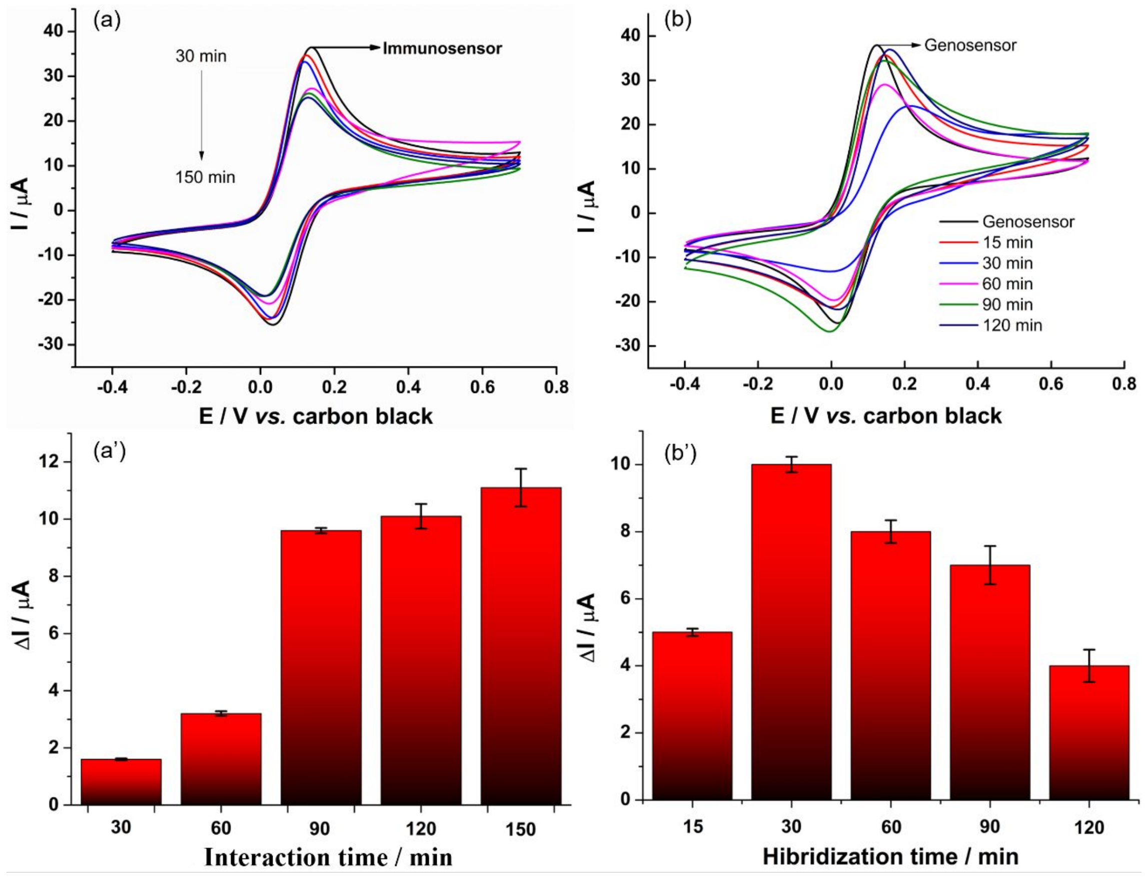

Fig. S8. Cyclic voltammograms to optimize the interaction of antigen and hybridization of target DNA analytes with the surface of the immunosensor or genosensor, respectively. Voltammograms in the presence of 1.0 mmol L^{-1} FcMeOH in 0.1 mol L^{-1} KCl (a) immunosensor varying the interaction time from 30 to 150 min, at antigen concentration of 0.5 µmol L^{-1}. (b) Genosensor varying hybridization time from 15 to 120 min, at target DNA concentration 10.0 µmol L^{-1}. (a'-b') Bar graph for analytical response of the

analytes (I_{pa}) of interest as a function of time variation. Scan rate: 50 mV s^{-1}. Responses obtained from the variation of the anode peak current.

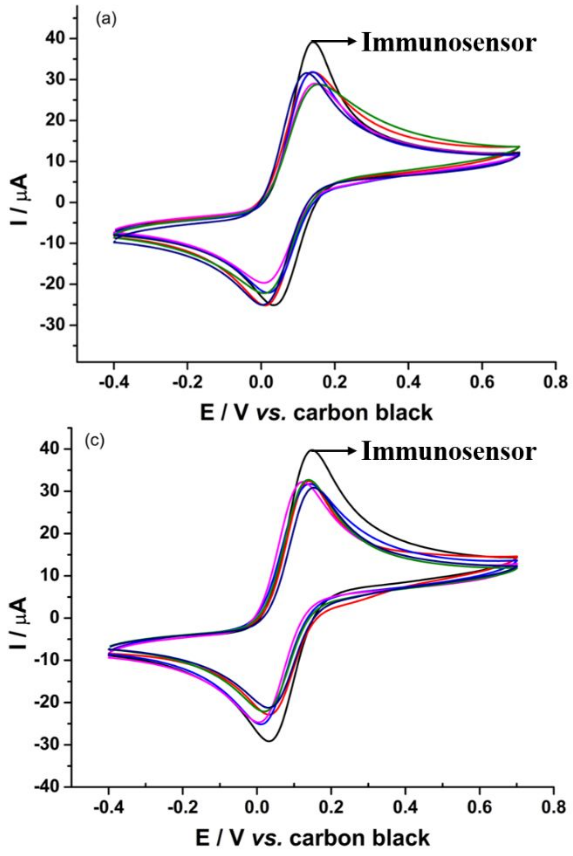

Fig. S9. Cyclic voltammograms obtained with the biosensors in the presence of 1.0 mmol L^{-1} FcMeOH in 01. mol L^{-1} KCl. (a-b) reproducibility tests for the (a) immunosensor and (b) genosensor. (c-d) repeatability tests for the (c) immunosensor and (d) genosensor). Scan rate 50 mV s^{-1}.

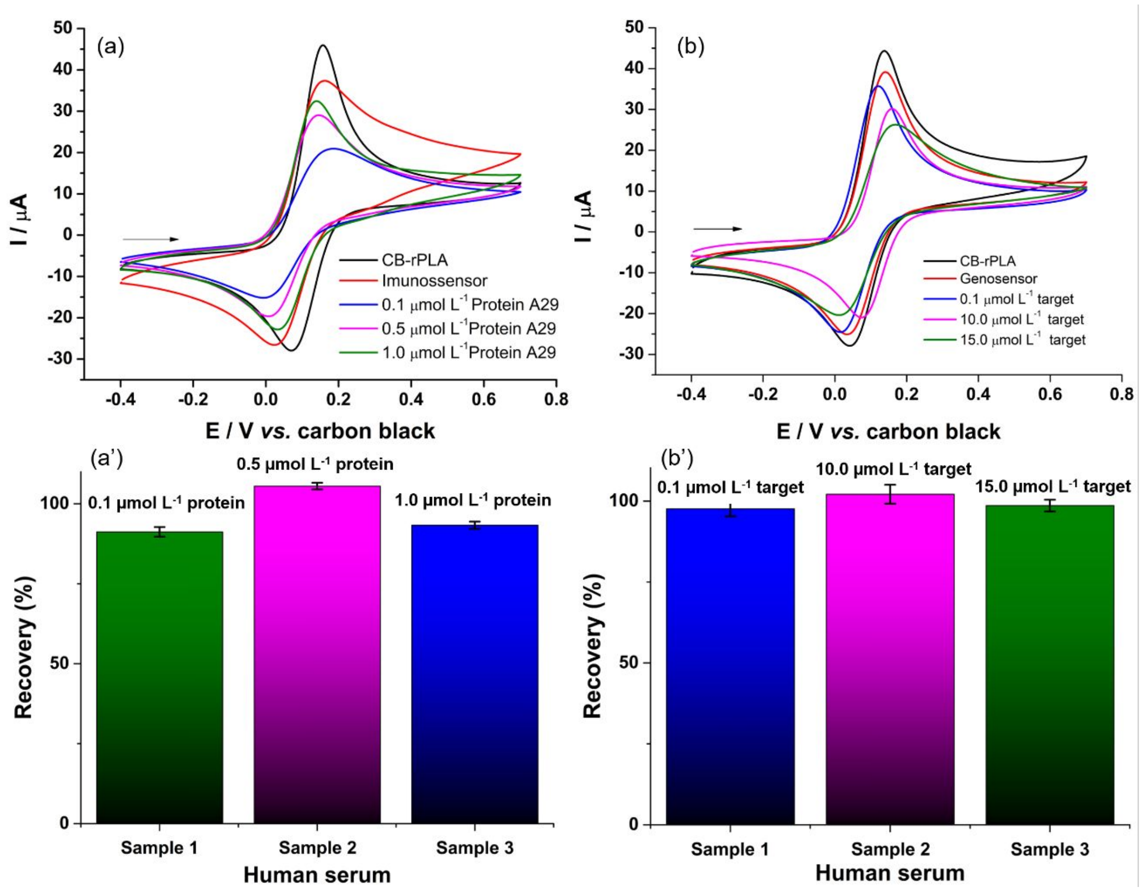

Fig. S10. Cyclic voltammograms obtained for the analysis of human serum samples spiked with known concentrations of MKPV target antigen (protein A29) and DNA. Responses obtained in the presence of 1.0 mmol L^{-1} FcMeOH in 01. mol L^{-1} KCl. (a1) immunosensor: antigen concentration of 1.0; 0.5 and 0.1 μmol L^{-1}. (b1) genosensor: DNA target concentration 0.1; 10.0 and 15.0 μmol L^{-1}. Scan rate 50 mV s^{-1}. Responses obtained from the variation of the anode peak current.

Table S1. Summary of optimized parameters for immunosensor and genosensor production.

Parameters	Immunossensor	Genosensor	Immunossensor	Genosensor
	Range studied		Chosen value	
**Antibody (concentration)**	0.5 – 20.0 μg L^{-1}	–	1.0 μg L^{-1}	–
**Antibody (time)**	30 – 150 min	–	60 min	–
**Interaction (time)**	30 – 150 min	–	90 min	–
**Capture DNA (concentration)**	–	1.0 – 10.0 μmol L^{-1}	–	6.0 μmol L^{-1}
**Capture DNA (time)**	–	15 – 105 min	–	15 min
**Hibridization (time)**	–	15 – 120 min	–	30 min

Table S2. Addition and recovery test on spiked human serum samples.

Sample	Immunossensor	Genosensor	Immunossensor	Genosensor
	Spiked (μmol L^{-1})		Recovery (%)	
**Human serum**	0.1	0.1	92 ± 5	98 ± 5
**Human serum**	0.5	10.0	104 ± 4	100 ± 7
**Human serum**	1.0	15.0	95 ± 4	98 ± 4
